# HIV Vaccine Development in the Aftermath of the STEP Study: Re-Focus on Occult HIV Infection?

**DOI:** 10.1371/journal.ppat.1000114

**Published:** 2008-08-29

**Authors:** Klaus Überla

**Affiliations:** Department of Molecular and Medical Virology, Ruhr-University Bochum, Bochum, Germany; The Scripps Research Institute, United States of America

Only two HIV vaccines have been taken through efficacy trials so far. In the first HIV vaccine efficacy trial started ten years ago, recombinant gp120 protein, the CD4-binding subunit of the HIV envelope, was used as vaccine antigen [1]. The vaccine neither prevented HIV acquisition nor reduced the viral load in those acquiring HIV infection. Although the vaccine was able to induce antibodies to gp120, these did not neutralize field isolates of HIV. Differences in the conformation between the monomeric gp120 subunit of the vaccine and the functionally active trimeric envelope spike on the surface of virus particles, HIV diversity, as well as various antibody escape mechanisms of the HIV envelope (reviewed in [2]), have been proposed to explain the inefficacy of the antibody-based gp120 vaccine. Given the difficulties of antibody-based HIV prevention strategies, the second HIV efficacy trial, the STEP study, tested whether the second arm of the adaptive immune response, cytotoxic T cells, would be able to provide protection. To induce cytotoxic T cell responses, replication-deficient adenoviral vectors transfering the *gag*, *pol*, and *nef* genes of HIV were used. Since all the three vaccine antigens used in this study are intracellular proteins that are usually not expressed on the surface of HIV-infected cells or HIV particles, vaccine-induced HIV-specific antibodies should not be able to contribute to protection. Thus, the study was specifically designed to explore the efficacy of HIV-specific cytotoxic T cells. A total of 3,000 volunteers with a high risk of acquiring HIV infection were either immunized three times intramuscularly with replication-deficient adenoviral vectors transfering the *gag*, *pol*, and *nef* genes of HIV, or received a placebo. As observed in non-human primate studies and previous phase I clinical trials, the adenoviral vector vaccine induced substantial HIV-specific cytotoxic T cell responses in most of the vaccinees [3]. However, at a planned interim analysis, 19 individuals in the vaccine arm and 11 individuals of the placebo arm acquired HIV infection during a follow-up of approximately 620 person years in both groups [4]. Incidences of 3.07 and 1.77 per 100 volunteers in the vaccine and placebo group, respectively, indicate that there was no beneficial effect of the vaccine on HIV acquisition.

**Figure 1:**
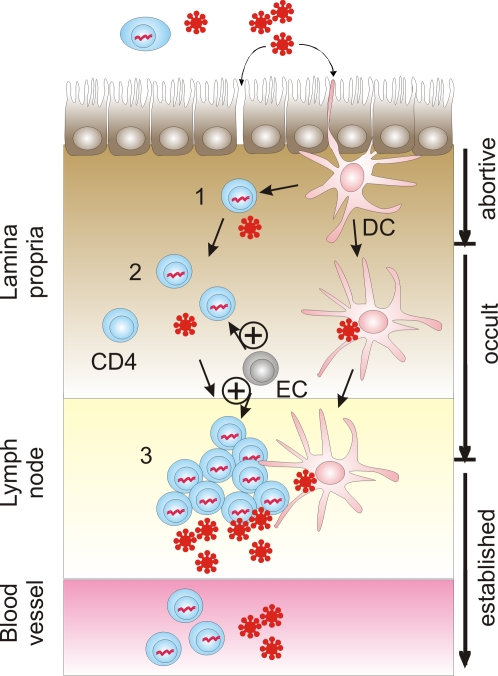
Model of the Early Stages of Mucosal HIV Infection (Modified from [11]) and Vaccine-Induced Enhancement of Infection. Free virus crosses the epithelial barrier of the mucosa through breaks or by transport on dendritic cells (DC), transcytosis, or infection of intraepithelial DC, macrophages, or CD4+ T cells. Initially, this will lead to a single HIV-infected cell (1) located in the lamina propria. Further spread can be blocked by infection of the first cell with a replication-deficient virus mutant, integration into a transcriptionally silent genomic region, or absence of susceptible secondary target cells, leading to abortive infection once the infected cell dies. If the virus is transmitted to secondary target cells (2), occult infections can occur if the reproductive rate of HIV-infected cells is reduced to less than one. These occult HIV infections are reported to be associated with detectable levels of HIV-specific cellular immune responses and can be defined by transient detection of virus in the absence of subsequent seroconversion. Transient viremia suggests that occult infections can still occur after spread of the virus to the regional lymph node (3). As outlined in the text, enhancement of the incidence of established, seropositive HIV infections in a subgroup of vaccinated volunteers of the STEP study could be explained by vaccine-induced, HIV-specific enhancer cells (EC) promoting virus spread by acting on secondary target cells (2) and/or a localized nidus of infection in the draining lymph node (3). If the enhancer cells indeed increase the number of established HIV infections by acting at stage 2 or 3, early HIV infections must resolve spontaneously in the absence of the vaccine-induced enhancer cells. An approximate 2-fold increase in the incidence of seropositive HIV infections in the vaccine subgroup compared to the placebo subgroup therefore suggests that less than half of all stage 2 or 3 infections in the placebo subgroup progress to established infections.

The HIV virus particle transmitted to an individual cannot be targeted by the vaccinees' cytotoxic T cells, because they require presentation of HIV-derived peptides on autologous MHC-I molecules. When looking at the different stages in the establishment of HIV infection after mucosal exposure (Figure 1), the earliest stage cytotoxic T cells could exert their beneficial effect is the killing of the first HIV-infected cell, presumably in the lamina propria of the exposed mucosa. However, given the low density of T cells in this compartment, it seems highly unlikely that an HIV-specific cytotoxic T cell encounters this single HIV-infected cell. Rather, it can be assumed that additional replication cycles and local spread of the virus or virus-infected cells to the draining lymph nodes occur prior to encounter with HIV-specific T cells. Subsequent activation and expansion of the HIV-specific T cells might be too slow to prevent further spread of the virus. Thus, rather then preventing HIV infection, the benefit of the cytotoxic T cells might be the reduction of viral load. However, the interim analysis of the STEP study also failed to provide any evidence for lower viral loads in the vaccine group [4]. Therefore, neither non-neutralizing gp120-specific antibodies nor HIV-specific cytotoxic T cells induced by the adenoviral vector vaccine were sufficient to provide protection.

The incidence of HIV infections in the vaccine group seemed to be higher than in the placebo group. This raised the critical question of whether vaccination actually enhances the frequency of HIV acquisition. Post-hoc analyses of different subgroups indicated that the incidence of HIV infections in volunteers with pre-existing humoral immunity to adenovirus prior to immunization was 2.3-fold (95% confidence interval 1.1 to 4.7) higher than in the respective placebo subgroup [4]. In contrast, there was no difference in the incidence of HIV infection between the vaccine and the placebo groups in the absence of pre-existing antibodies to adenovirus. An initial comparison of the distribution of risk factors in the vaccine and placebo subgroups with high levels of pre-existing adenoviral immunity revealed a good match of baseline variables such as location, race, age, risk behavior, circumcision, and history of sexual transmitted diseases [5]. If this holds up for other confounding factors, the enhanced incidence of HIV infections in this vaccinated subgroup might have severe implications for all subsequent HIV vaccine trials, as well as for vaccine and gene therapy trials using adenoviral vectors. It is therefore important to explore how such a vaccine could increase the susceptibility to HIV infection. Theoretically, this could be either due to an excessive adenovirus-specific immune reaction or due to the induction of detrimental HIV-specific immune responses.

Injection of the adenoviral vector particle can induce an immediate innate response leading to increased inflammatory cytokine levels in the blood, which return to baseline levels within 3 to 7 days [6]. However, since the increased HIV incidence in the vaccine subgroup with pre-existing adenoviral immunity seems to persist for at least a year [4], the immediate innate response is unlikely to be responsible for the observed increase in susceptibility to HIV infection in the vaccine subgroup. Injection of the adenoviral particle into individuals with pre-existing adenoviral immunity also raises a recall response, including activation of adenovirus-specific CD4+ T cells. These activated CD4+ T cells could serve as additional target cells for HIV in the lamina propria and therefore enhance the risk of HIV infection. However, given the vast amount of antigens and infections humans are continuously exposed to, it seems highly unlikely that intramuscular injection of the adenoviral particle can notably raise the number of susceptible CD4+ T cells in the rectal or male genital mucosa above their pre-existing background level for an extended period. Another argument against this hypothesis is that the percentage of adenovirus-specific CD4+ T cells in the blood was actually lower in the group with pre-existing immunity to adenovirus [3].

Thus, it seems more plausible that HIV-infected cells or HIV antigen presenting cells (stages 2 and 3 in Figure 1) activate vaccine-induced, HIV-specific T cells in the lamina propria of the exposed mucosa or in its draining lymph node. In the absence of effective antiviral effector mechanisms, these activated HIV-specific “enhancer” T cells could favour early spread of the HIV infection. Whether this is only a risk associated with adenoviral vector vaccines encoding HIV antigens or is also shared by other HIV vaccines is unclear. However, two characteristics of the vaccine used in the STEP study suggest a note of caution to premature generalization. First, the enhancement of HIV infection is only detected in the presence of pre-existing adenoviral immunity. Thus, the injected adenoviral vector particle might trigger regulatory events, which also suppress or modulate as a bystander effect the HIV-specific T cell responses induced by the adenoviral vector-encoded HIV vaccine antigens. Second, the absence of an Env component might delay the sensing of the HIV infection by antibodies (even non-neutralizing ones) at an early infection stage, thereby preventing timely recruitment of vaccine-induced effector mechanisms to the early replication sites of HIV. As neither humoral nor cellular immune responses alone seem to be sufficient for protection from HIV, vaccines inducing both effector arms need to be evaluated. It seems too early to dispense with the potential of adenoviral vector-based HIV vaccines since they are among the most efficient inducers of cytotoxic T cell responses in humans and a large number of animal models.

Whatever the precise mechanism is that led to enhanced acquisition of HIV infection in the STEP study vaccine group with pre-existing adenovirus immunity, it most likely acts at a stage subsequent to infection of the first cell in the recipient individual. The barrier function of the mucosal epithelia should not be affected by the intramuscular vaccination. The number of HIV-susceptible cells in the lamina propria prior to HIV exposure should not be enhanced above the pre-existing background level given the continous exposure to antigens and frequent infections with all kinds of pathogens and commensals. Since all the vaccine antigens used in the STEP study are intracellular proteins and usually not expressed on the surface of HIV-infected cells or HIV particles, only infected recipient cells, but not the initial virus particle leading to infection, can trigger the vaccine-induced enhancer cells to mediate their enhancing effect.

A likely candidate for these enhancer cells are vaccine-primed CD4+ T cells re-activated by HIV peptides presented on MHC molecules of antigen presenting cells and/or HIV-infected cells. Since encounter of a vaccine-induced, HIV-specific enhancer cell and the first infected recipient cell seems to be an unlikely event, the enhancement of infection probably acts at one of the subsequent rounds of infection. It is worth noting that vaccination did not notably enhance viral load in infected individuals but enhanced the incidence of infected persons as diagnosed by seroconversion. If the vaccine does not enhance infection at the first stage, it must enhance the frequency with which vaccinees progress through subsequent stages. This implies that some unvaccinated individuals must be able to control transition from the early stages of infection to later ones. In the absence of seroconversion, infections that are contained at these early stages are not diagnosed.

Evidence for the existence of such occult immunodeficiency virus infections has come so far from frequently exposed seronegative individuals (reviewed in [7],[8]) and immunodeficiency virus–exposed non-human primates [9],[10]. In the latter animal models, the monkeys remain seronegative despite detection of simian immunodeficiency virus–specific T cell responses. Furthermore, low levels of viral RNA can be detected only transiently in the blood of the exposed animals, indicating that control of immunodeficiency virus spread is possible even after production of substantial amounts of virus particles. Assuming an unbiased distribution of risk factors in the vaccine and placebo subgroups with pre-existing adenoviral immunity, the results of the STEP study provide further support for the occurrence of occult HIV infections in humans and even allow an estimation of the lower limit of the frequency of occult HIV infections. An approximate 2-fold increase in the incidence of seropositive HIV infections in the vaccine subgroup with pre-existing adenoviral immunity suggests that at least half of all early stage HIV infections of the non-immunized study participants with pre-existing adenoviral immunity are contained prior to seroconversion. If the enhancing effect of vaccination does not mediate progression of all early stage HIV infections into established HIV infections, the ratio of occult infections to evident infections should even be larger than two. Therefore, occult HIV infections seem to be much more frequent than generally assumed. A better understanding of the determinants of occult infections is urgently needed and might provide novel strategies for HIV vaccine development.
